# A therapeutic HBV vaccine containing a checkpoint modifier enhances CD8^+^ T cell and antiviral responses

**DOI:** 10.1172/jci.insight.181067

**Published:** 2024-11-08

**Authors:** Mohadeseh Hasanpourghadi, Mikhail Novikov, Robert Ambrose, Arezki Chekaoui, Dakota Newman, ZhiQuan Xiang, Andrew D. Luber, Sue L. Currie, XiangYang Zhou, Hildegund C.J. Ertl

**Affiliations:** 1 The Wistar Institute, Philadelphia, Pennsylvania, USA.; 2 Virion Therapeutics, Newark, Delaware, USA.

**Keywords:** Immunology, Vaccines, Hepatitis, Immunotherapy, T cells

## Abstract

In patients who progress from acute hepatitis B virus (HBV) infection to a chronic HBV (CHB) infection, CD8^+^ T cells fail to eliminate the virus and become impaired. A functional cure of CHB likely requires CD8^+^ T cell responses different from those induced by the infection. Here we report preclinical immunogenicity and efficacy of an HBV therapeutic vaccine that includes herpes simplex virus (HSV) glycoprotein D (gD), a checkpoint modifier of early T cell activation, that augments CD8^+^ T cell responses. The vaccine is based on a chimpanzee adenovirus serotype 6 (AdC6) vector, called AdC6-gDHBV2, which targets conserved and highly immunogenic regions of the viral polymerase and core antigens fused to HSV gD. The vaccine was tested with and without gD in mice for immunogenicity, and in an AAV8-1.3HBV vector model of antiviral efficacy. The vaccine encoding the HBV antigens within gD stimulates potent and broad CD8^+^ T cell responses. In a surrogate model of HBV infection, a single intramuscular injection achieved pronounced and sustained declines of circulating HBV DNA copies and HBV surface antigen; both inversely correlated with HBV-specific CD8^+^ T cell frequencies in spleen and liver.

## Abstract 2

The manuscript describes a vaccine that in pre-clinical studies decreased viral loads in a mouse model of chronic hepatitis B virus infection

## Introduction

The control of hepatitis B virus (HBV) infections is mainly mediated by CD8^+^ T cells (1, 2), which are only poorly induced in the 5%–10% of patients unable to clear the infection, resulting in continued virus replication (3). T cell activation in response to antigens within the liver is, in general, attenuated due to low expression of costimulators, enhanced levels of inhibitory factors such as soluble mediators (e.g., arginase, indoleamine 2,3-dioxygenase [IDO]) and antiinflammatory cytokines (e.g., IL-10 and TGF-β), regulatory T (Treg) cells, and myeloid-derived suppressor cells (4–8). Once T cells are activated, the high and sustained levels of HBV antigens, which are in part derived from covalently closed circular DNA (cccDNA) minichromosomes (9) or integrated DNA (10), drive their exhaustion, which impacts their ability to proliferate or exert effector functions and eventually causes their death (4, 11).

Current therapies that are being developed for functional cure of chronic HBV (CHB) infection defined by the FDA as sustained undetectable circulating HBV surface antigen (HBsAg) and HBV DNA, or HBV DNA alone, after a finite course of treatment (12), are predominantly focusing on viral targets to prevent replication by nucleos(t)ide reverse transcriptase inhibitors or core assembly modulators (13, 14), inhibit gene expression by, for example, small interfering RNAs (siRNAs) or antisense oligonucleotides (15–17), or transfer or induction of antibodies to remove circulating HBsAg with the goal to restore immune control upon treatment discontinuation (18). These interventions, when used alone or in combination, fail to reliably produce functional cures (19, 20). Immune-based interventions that increase HBV-specific CD8^+^ T cell responses have mainly targeted already-activated and functionally impaired CD8^+^ T cells through checkpoint inhibitors or by expanding them by vaccines (21–24). The concept of restoring immune control by rescuing the same immune responses that failed previously, especially after decades of ongoing viral antigen exposure, may be curbed by the limited number of remaining CD8^+^ T cells that can still respond (25). For example, investigational vaccine combination regimens that include anti–PD-1 antibodies and pegylated interferon (IFN) regimens have predominantly shown off-treatment HBsAg declines or occasional functional cures upon treatment discontinuation in younger patients and those with lower baseline HBsAg levels, both factors suggestive of residual immune functions (26–28).

To optimize CD8^+^ T cell responses to HBV, we developed AdC6-gDHBV2, a replication-defective chimpanzee adenovirus (AdC) vector that expresses highly immunogenic and conserved sequences from HBV core and polymerase (Pol). As high, but not low, levels of antigen present for a prolonged time trigger terminal exhaustion of responding CD8^+^ T cells (29), we chose parts of these 2 antigens rather than of HBsAg, which is expressed at excessive levels in patients with CHB (30, 31) and may drive exhaustion of specific CD8^+^ T cells. This notion is supported by the finding that in patients with CHB, CD8^+^ T cells against epitopes of the envelope protein, unlike those against epitopes of core or Pol, fail to regain functions after therapy with a checkpoint inhibitor (32). In addition, T cells against regions of HBV core and Pol have been associated with prevention of viral escape and/or hepatic flares upon antiviral discontinuation in patients with CHB (33–36). The vaccine antigen is expressed as a fusion protein within herpes simplex virus (HSV) glycoprotein D (gD), an intrinsic checkpoint modifier that enhances and broadens CD8^+^ T cell responses by blocking inhibition of early T cell activation by the B and T lymphocyte attenuator (BTLA), thereby lowering the T cells’ activation threshold (37, 38); this in turn enhances responses to dominant epitopes and allows for the activation of T cells against subdominant epitopes (36). The latter are generally not stimulated by a virus infection (39), so that even chronically infected individuals should still carry naive precursors. In addition, T cells against subdominant epitopes do not differentiate as rapidly toward exhaustion as the more potent T cells against immunodominant epitopes and they are more resistant to immunosuppression (40), which could result in better retention of functions in chronically infected hosts.

The purpose of our study was to evaluate the preclinical immunogenicity and efficacy of AdC6-gDHBV2, an intramuscularly (i.m.) administered vaccine that contains an inbuilt checkpoint modifier. Specifically, we wanted to confirm that gD could enhance and broaden responses of vaccine insert–specific CD8^+^ T cells and augment their ability to reduce HBV genome copies and HBsAg from mice with surrogate HBV infection.

## Results

### Vaccine immunogenicity.

**Figure 1:**
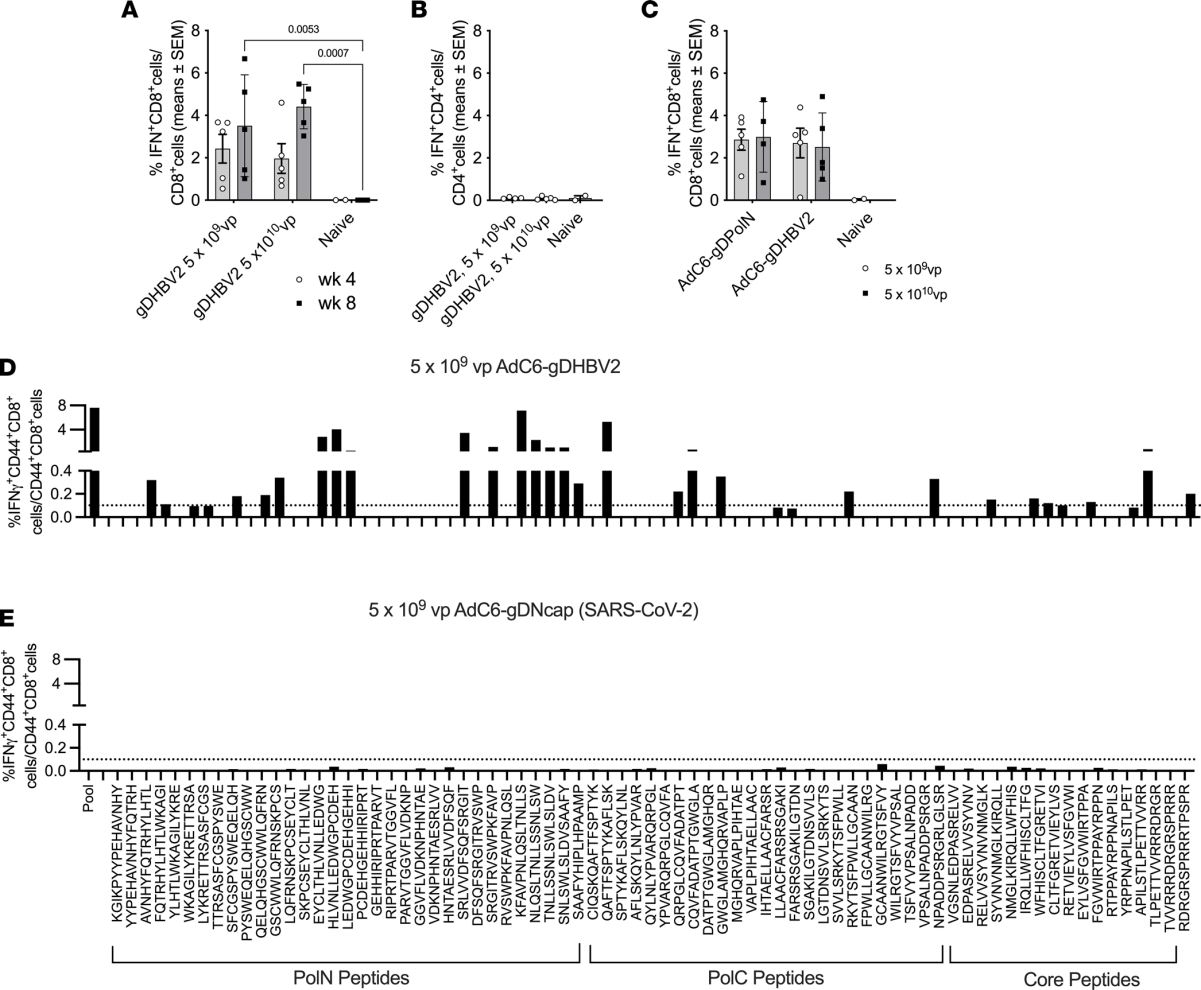
Vaccine immunogenicity. (**A**) Frequencies of circulating IFN-γ–producing CD8^+^ T cells responding to AdC6-gDHBV2 given at the indicated doses. T cells from 5 individual experimental mice and 2–4 naive control mice were analyzed 4 and 8 weeks after vaccination. The graph shows responses of individual animals, with bars indicating mean ± SEM. Significant differences are shown by connecting lines with *P* values above. (**B**) Data for CD4^+^ T cells from the same mice. (**C**) Frequencies of CD8^+^ T cells against AdC6-gDPolN and AdC6-gDHBV2. Responses were tested from blood of 5 individual mice 8 weeks after vaccination. Comparisons by 2-way ANOVA (**B** and **C**) showed no significant differences. (**D** and **E**) Frequencies of splenic CD44^+^CD8^+^ T cells versus all CD44^+^CD8^+^ T cells responding to the individual peptides representing the inserts derived from Pol and core were determined from pooled splenocytes of 5 mice. The *y* axis is identical for all graphs. Responses ≥0.1% indicated by the dotted line were viewed as positive.

To assess the immunogenicity of the AdC6-gDHBV2 vaccine, groups of 5 male C57BL/6 mice were immunized i.m. with 5 × 10^9^ or 5 × 10^10^ virus particles (vp) and their PBMCs were tested for CD8^+^ T cell responses 4 and 8 weeks later. AdC6-gDHBV2 induced potent CD8^+^ T cells responses at both doses that were stable between weeks 4 and 8 (Figure 1A), whereas CD4^+^ T cell responses tested at week 8 were marginal (Figure 1B). Next, we compared the immunogenicity of the AdC6-gDHBV2 vector to that of the previously described AdC6-gDPolN vector (41); groups of 5 male C57BL/6 mice were immunized i.m. with 5 × 10^9^ or 5 × 10^10^ vp of each vector and PBMCs were tested 8 weeks after vaccination. AdC6-gDPolN and AdC6-gDHBV2 induced similar CD8^+^ T cell responses (Figure 1C).

To determine the breadth of CD8^+^ T cell responses, pooled splenocytes were tested 4 weeks (5 × 10^9^ vp dose) after vaccination against the individual peptides that are part of the vaccines’ HBV insert (Figure 1D). To ensure the specificity of the assay, splenocytes from mice that had been injected i.m. with 5 × 10^9^ vp of an AdC6 vector expressing a fusion antigen of gD and the nucleocapsid protein of SARS-CoV-2 were tested upon peptide stimulation as well (Figure 1E). CD8^+^ T cells of AdC6-gDHBV2–immunized mice responded to multiple peptides within Pol and core, while the control vector failed to elicit a response to any of the peptides.

### The effect of gD on vaccine immunogenicity.

**Figure 2:**
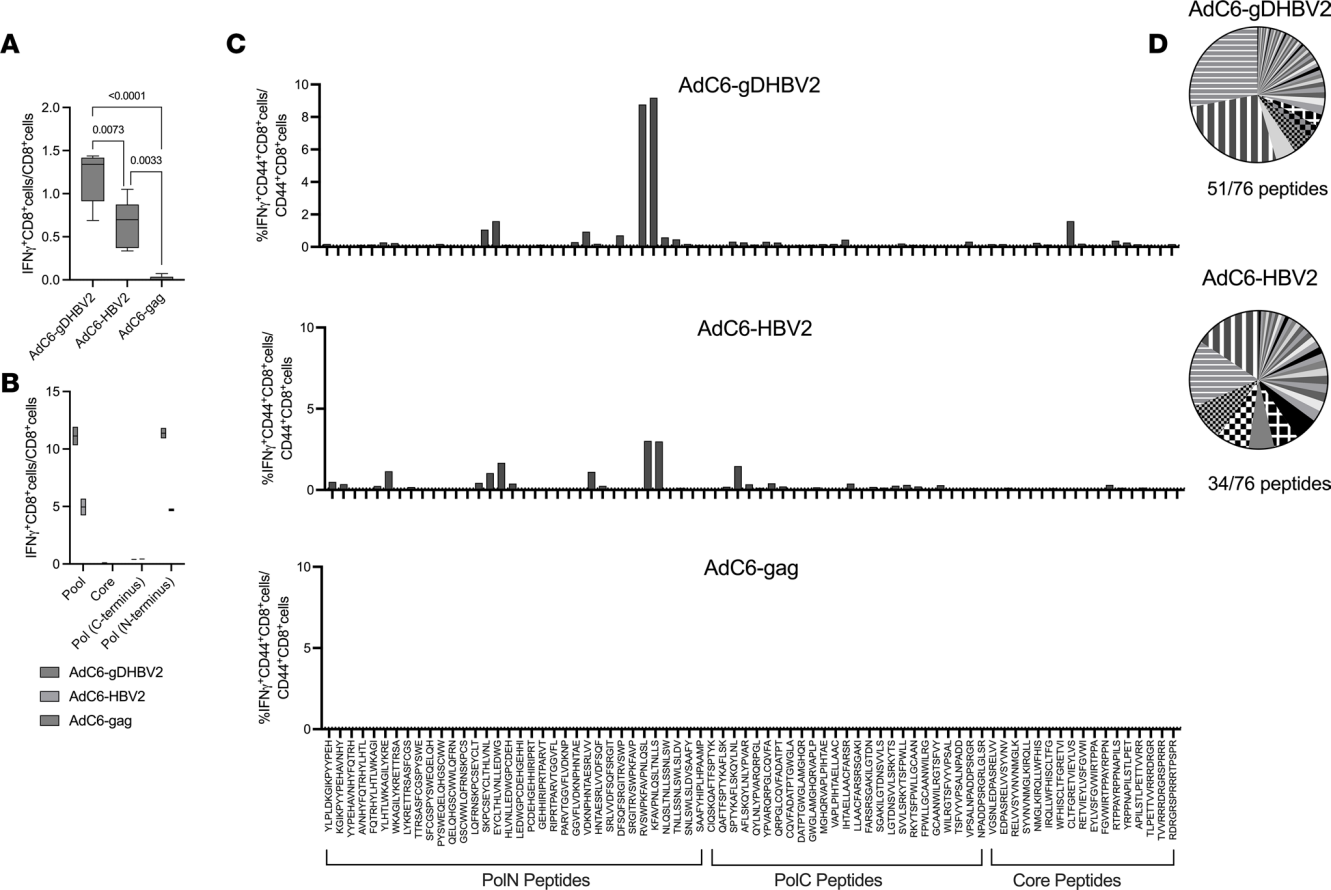
Effects of gD on vaccine immunogenicity. Mice were immunized with 1 × 10^10^ vp of AdC6 vectors expressing gDHBV2, HBV2, or gag of HIV-1. (**A**) PBMCs from 5 individual mice were tested 4 weeks later for responses to the HBV peptide pool. *P* values for differences between groups calculated by 2-way ANOVA are shown above the lines. (**B**) Duplicate samples of pooled splenocytes from 5 mice/group were tested 8 weeks after immunization for CD8^+^ T cell responses to peptides representing the HBV2 (pool), or N- and C-terminal sequences of Pol or core. (**C**) Splenocytes harvested 8 weeks after vaccination were tested for CD44^+^CD8^+^ T cell responses to individual peptides of the HBV2 sequence. (**D**) The pie charts show relative responses to individual peptides. Peptides that are strongly recognized by T cells from mice immunized with the AdC6-HBV2 or AdC6-gDHBV2 vaccine are highlighted by fill patterns.

To ensure that gD improves CD8^+^ T cell responses to the HBV2 insert, groups of C57BL/6 mice were injected i.m. with 1 × 10^10^ vp of AdC6-gDHBV2, AdC6-HBV2, and an AdC6 vector expressing HIV-1 gag (AdC6-gag) as a control. PBMCs were tested 4 weeks later by intracellular cytokine staining for frequencies of HBV2-specific CD8^+^ T cells. The presence of gD increased HBV2-specific CD8^+^ T cell frequencies approximately 2-fold (Figure 2A). Eight weeks after vaccination, splenocytes were tested for CD8^+^ T cells against the peptide pool of the HBV2 insert, or subpools reflecting the sequences of the N- and C-terminus of Pol or core. Responses to gDHBV2 compared with HBV2 were higher using the entire peptide pool or the peptide pool corresponding to the N-terminus of Pol (Figure 2B). Splenocytes were tested for responses to individual peptides (Figure 2, C and D). Responses to some peptides were comparable, while others were only elicited by the AdC6-gDHBV2 vaccine, which elicited a broader response than AdC6-HBV2 (51 vs. 34 out of 76 peptides) (Figure 2, D and E). These data confirm that gD increases and broadens CD8^+^ T cell responses to the HBV2 insert.

### Vaccine efficacy and T cell responses in HBV-carrying mice.

**Figure 3:**
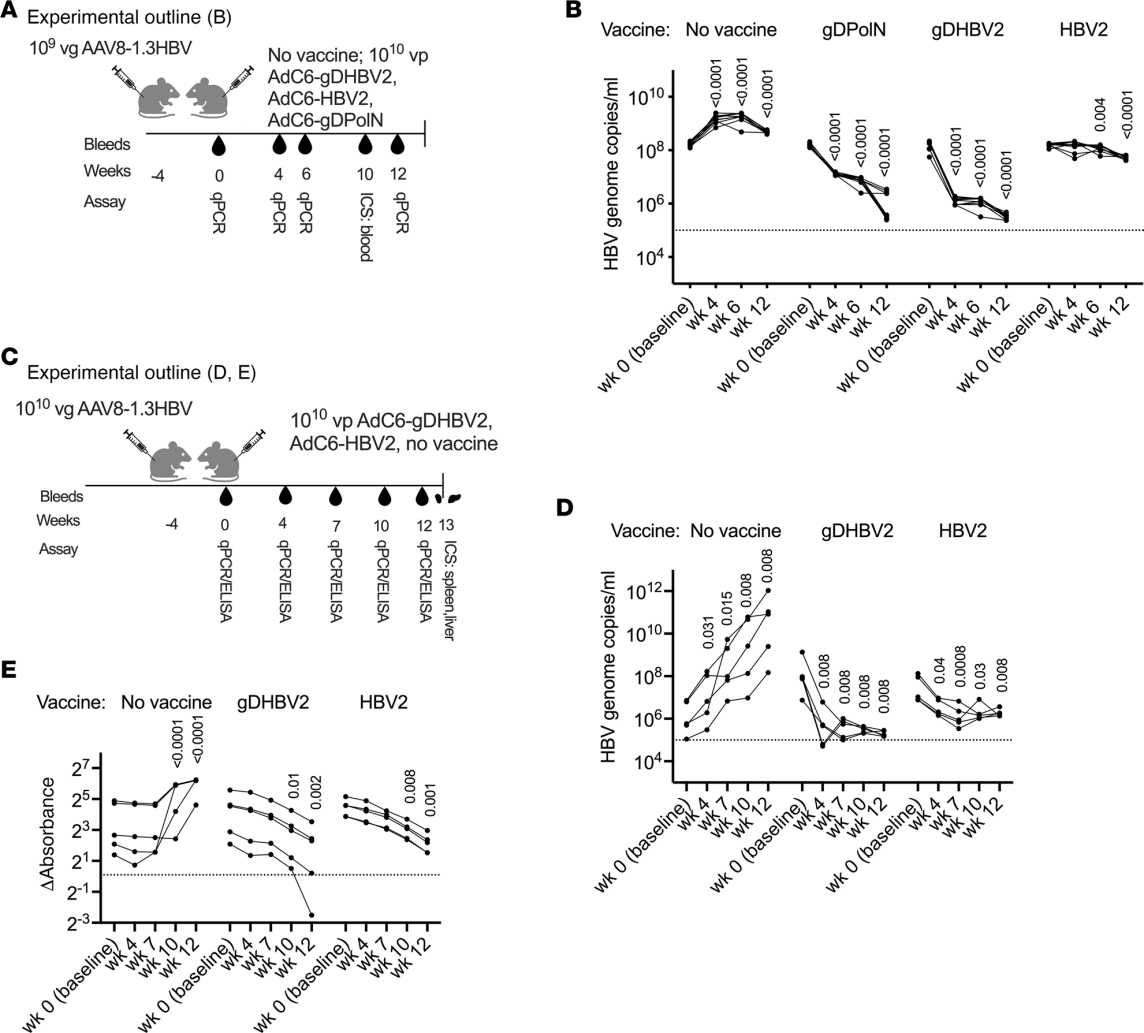
Vaccine efficacy in mice infected for 4 weeks with AAV8-1.3HBV. (**A**) Experimental outline for graphs in **B** and Figure 4, A and B for 10 mice that were injected with 1 × 10^9^ vg of AAV8-1.3HBV. (**B**) HBV genome copy levels in serum over time. Significant differences comparing baseline to after-vaccination data were calculated by multiple unpaired Mann-Whitney test and levels of significance are displayed within the graph. The dotted line at 10^5^ indicates the detection limit. (**C**) Experimental outline for graphs **D** and **E** and Figure 4, C–G for 5 mice that were injected with 1 × 10^10^ vg of AAV8-1.3HBV. (**D**) HBV genome copy levels in serum over time. Significant differences comparing baseline to after-vaccination data were calculated by multiple unpaired Mann-Whitney test and levels of significance are displayed on top of each group. The dotted line at 10^5^ indicates the detection limit. (**E**) ELISA data for HBsAg in sera are shown as absorbance values from which background data had been subtracted. The dotted line shows results for the negative control (serum from naive mice). Significant differences versus baseline were determined by 2-way ANOVA and *P* values are displayed within the graph.

To assess vaccine efficacy, mice were injected i.v. with 1 × 10^9^ viral genome copies (vg) of adeno-associated virus 8–1.3HBV (AAV8-1.3HBV) and vaccinated 4 weeks later i.m. (Figure 3A) with 1 × 10^10^ vp AdC6-gDPolN, AdC6-gDHBV2, AdC6-HBV2, or nothing. Serum HBV DNA copies were determined at baseline and then at different times after vaccination (Figure 3B). Before vaccination, high levels of circulating HBV genomes could be detected. In unvaccinated control mice, HBV genome copies increased by approximately 1 log_10_ copies/mL (cps/mL) within the next 4 weeks and remained stable or slightly declined thereafter. By week 4 after vaccination, AdC6-gDPolN–immune mice showed a 1 log_10_ cps/mL reduction in viral loads compared with baseline and this difference increased to approximately 2 log_10_ cps/mL by week 12. AdC6-gDHBV2 reduced viral loads more rapidly and achieved by week 4 a 2 log_10_ cps/mL decrease, with further declines by week 12. The AdC6-HBV2 vaccine was less efficacious and achieved only a 1 log_10_ cps/mL reduction overall.

The experiment was repeated with a higher challenge dose of 1 × 10^10^ vg of AAV8-1.3HBV (Figure 3C). HBV genome copies in unvaccinated control mice increased from baseline to week 12 by 4.9 log_10_ cps/mL (Figure 3D). Mice immunized with AdC6-gDHBV2 achieved by week 12 after vaccination a significant viral load reduction of 3.2 log_10_ cps/mL from baseline, while mice vaccinated with AdC6-HBV2 achieved a 1.4 log_10_ cps/mL reduction from baseline. Levels of HBsAg were tested from sera and showed increases in control mice and significant decreases by week 12 after vaccination in AdC6-gDHBV2– and AdC6-HBV2–vaccinated animals (Figure 3E).

**Figure 4:**
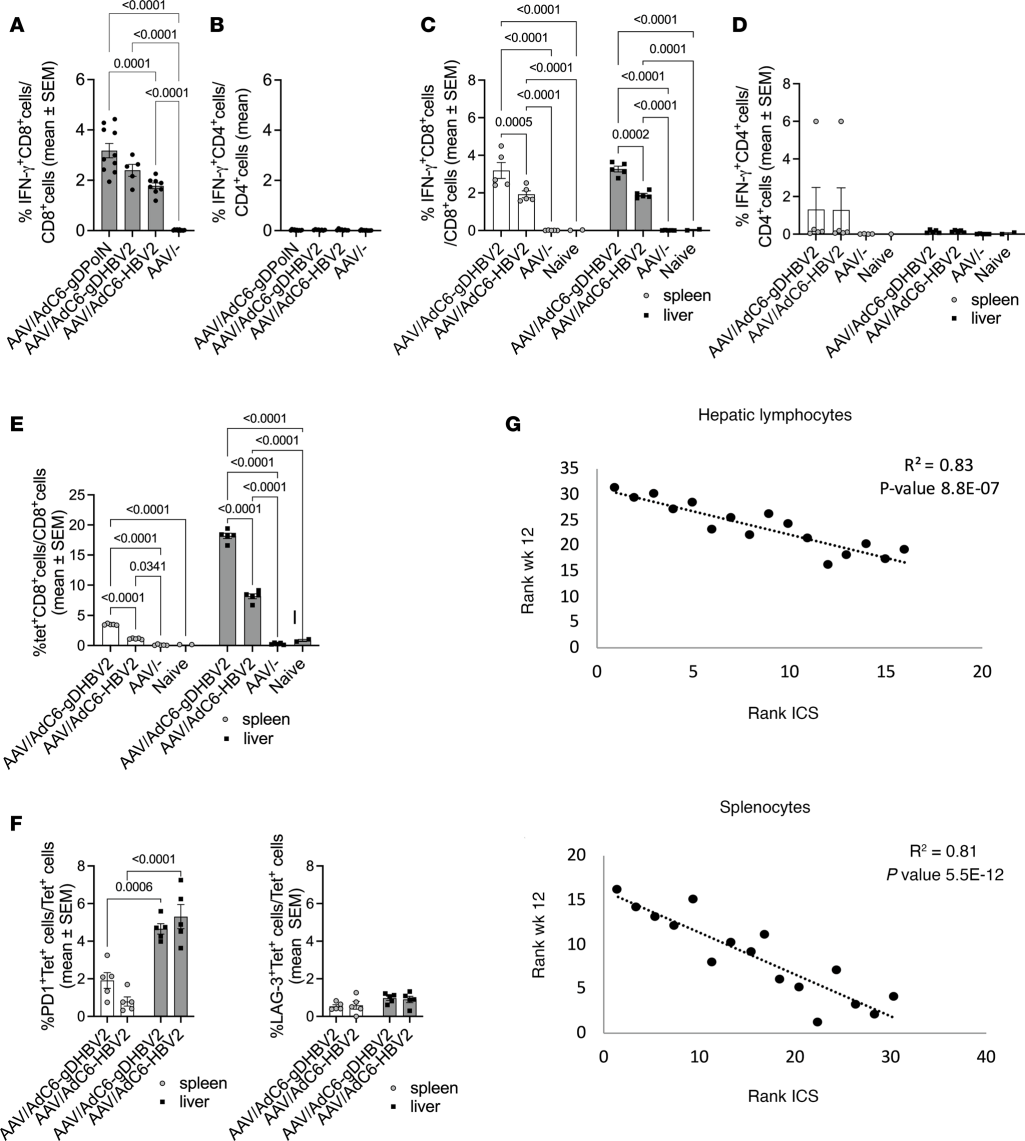
Vaccine immunogenicity in AAV8-1.3HBV–infected mice. (**A** and **B**) Experimental outline shown in Figure 3A. (**A**) Frequencies of IFN-γ–producing CD8^+^ T cells or (**B**) CD4^+^ T cells in blood were tested for 5–10 mice 10 weeks after vaccination. Significant differences with *P* values are displayed on lines above graphs. (**C**–**G**) Experimental outline shown in Figure 3C. (**C**) Frequencies of IFN-γ–producing CD8^+^ T cells or (**D**) CD4^+^ T cells in spleens and livers of 5 experimental mice and 2 control mice 12 weeks after vaccination. *P* values shown above the lines were calculated by 2-way ANOVA. (**E**) Frequencies of tetramer^+^ (tet^+^) CD8^+^ T cells in spleens and livers of 5 experimental mice and 2 naive control mice injected with 1 × 10^10^ vg of AAV8-1.3HBV, vaccinated 4 weeks later with 1 × 10^10^ vp of the indicated vectors or nothing, and tested 12 weeks later. *P* values calculated by 2-way ANOVA are shown above the lines. (**F**) Percentage of tet^+^CD8^+^ cells of 5 mice per group expressing PD-1 or LAG-3. Gates were set based on expression on resting CD44^–^CD8^+^ cells. *P* values calculated by 2-way ANOVA are shown above the lines. (**G**) Spearman’s correlations between frequencies of IFN-γ-producing HBV-specific CD8^+^ T cells in spleens or livers and viral loads based on intracellular cytokine staining (ICS) of 15 samples. *R*^2^ and *P* values are shown in the upper right corner of the graphs.

In the first experiment (Figure 3A), mice were bled 10 weeks after vaccination and PBMCs were tested for HBV-specific T cells. Mice immunized with AdC6-gDHBV2 and AdC6-HBV2 developed significant frequencies of HBV-specific CD8^+^ T cells (Figure 4A), while CD4^+^ T cell responses were negative (Figure 4B).

T cells from spleens and livers were tested in the second experiment (Figure 3C). CD8^+^ T cell responses were positive in both tissues and significantly higher in AdC6-gDHBV2– than AdC6-HBV2–vaccinated mice (Figure 4C). Only 1 vaccinated mouse developed low but detectable HBV-specific CD4^+^ T cell responses in spleen (Figure 4D). Lymphocytes were also tested by staining with a tetramer for an immunodominant epitope present within the Pol sequence (Figure 4E). Both vaccines induced significant CD8^+^ T cell responses. Frequencies of tetramer-positive cells were higher in livers than spleens, and the AdC6-gDHBV2 vaccine achieved significantly higher responses in both tissues compared with AdC6-HBV2 (Figure 4E). Cells were concomitantly stained for 2 exhaustion markers (Figure 4F), i.e., PD-1 and LAG-3 (Figure 4F). Frequencies of tetramer^+^ CD8^+^ T cells that were positive for these markers were low, indicating that T cells had likely not become exhausted.

Frequencies of HBV-specific CD8^+^ T cells in spleens or livers inversely correlated with HBV genome copy numbers (Figure 4G). We tested for other correlations and as expected, viral loads showed positive correlations for different time points after vaccination and with HBsAg levels at week 12, while immune responses in different tissues, or tested by different methods, showed positive correlations with each other and inverse correlations with viral loads and HBsAg levels by week 12 after vaccination (Supplemental Figure 1A; supplemental material available online with this article; https://doi.org/10.1172/jci.insight.181067DS1).

The AAV8-1.3HBV vector given i.v. failed to stimulate detectable frequencies of CD8^+^ T cells, which were induced upon its i.m. but not i.v. injection. Many of the T cells induced by the i.m. injection of AAV8-1.3HBV responded to the same peptides that were recognized by AdC6-gDHBV2–induced CD8^+^ T cells, indicating that they should detect their targets on AAV8-1.3HBV–infected cells (Supplemental Figure 1B).

At 12 weeks after vaccination, mice were tested for antibodies against HBsAg. Anti-HBsAg antibodies were detected at low levels that failed to reach significance in AdC6-gDHBV2–vaccinated mice; no antibodies were detected in mice that only received the AAV8-1.3HBV vector (Supplemental Figure 1C).

**Figure 5:**
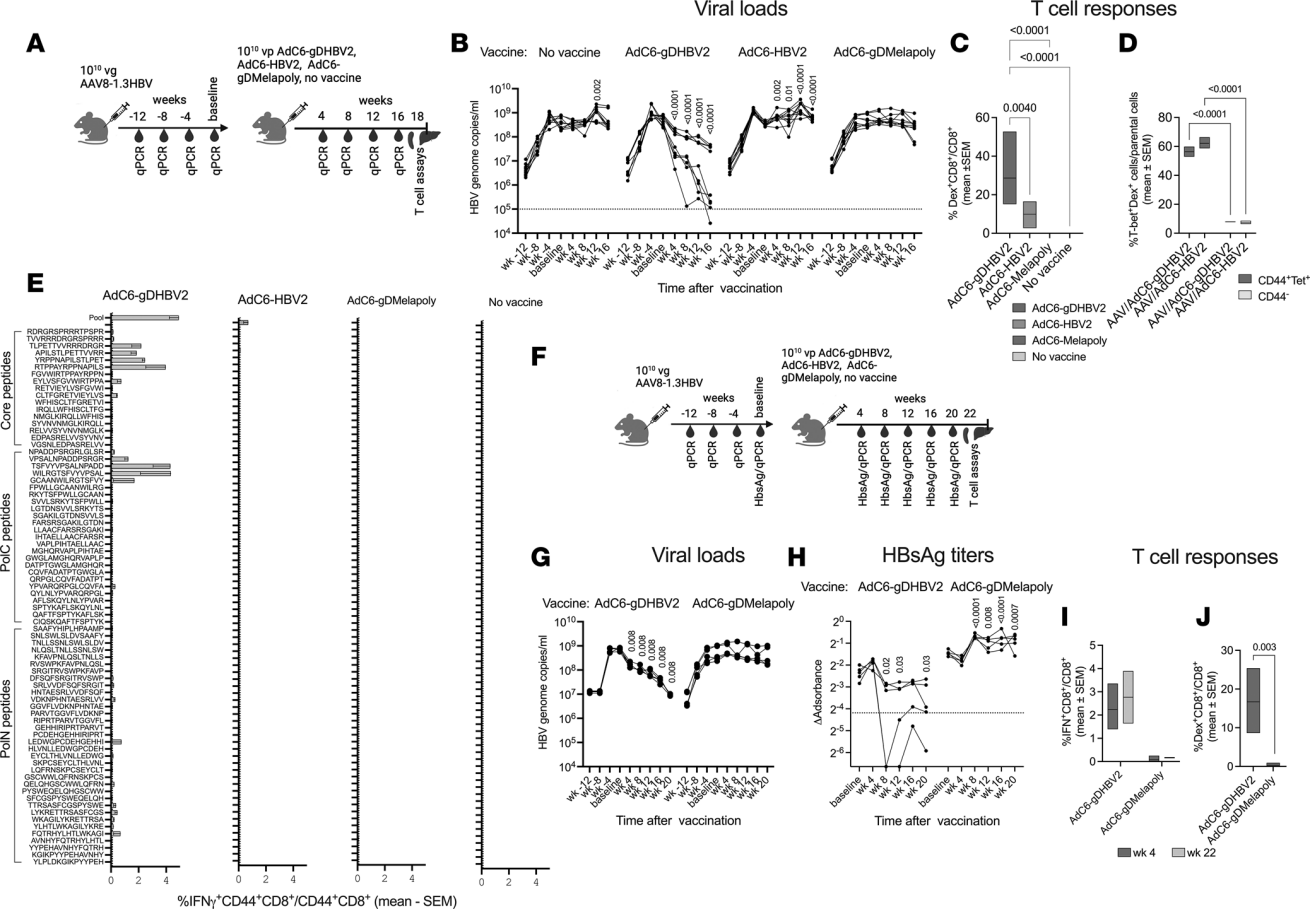
Vaccine efficacy in mice infected for 16 weeks with AAV8-1.3HBV. (**A**) Experimental outline for graphs in **B**–**E**. (**B**–**E** and **G**–**K**) Mice (*n* = 10) were injected with 1 × 10^10^ vg of AAV8-1.3HB. They were vaccinated 16 weeks later with the indicated vaccines. (**B**) Viral loads before and after vaccination shown as in Figure 3. Data were analyzed by multiple unpaired Mann-Whitney test against data obtained at baseline. *P* values are shown within the graphs. (**C**) Frequencies of CD8^+^ T cells from liver tested with a dextramer (dex) specific for an epitope within Pol from 5–10 mice. Data were analyzed by 1-way ANOVA and *P* values are shown above the lines. (**D**) Frequencies of AdC6-gDHBV2– or AdC6-HBV2–induced dex^+^CD44^+^CD8^+^ or dex^+^CD44^–^CD8^+^ liver lymphocytes of 5 mice per group that stained positive for T-bet. (**E**) Frequencies of CD44^+^CD8^+^ splenocytes tested against individual peptides representing the HBV2 insert and against the peptide pool. Data obtained without peptide stimulation were subtracted. (**F**) Experimental design for graphs in **G**–**K** based on experiments conducted with 5 mice per group. (**G**) Viral loads before and after vaccination shown as in Figure 3. Data were analyzed by multiple unpaired Mann-Whitney test against data obtained at baseline. Significant *P* values are shown within the graphs. (**H**) HbsAg levels are shown as OD values from which background values had been subtracted. The dotted line indicates results obtained with naive sera. Data were analyzed by Fisher’s LSD test comparing postvaccination time points to baseline; *P* values are shown within the graph. (**I**) PBMCs (week 4, *n* = 5) and spleens (week 22, *n* = 2) were tested by intracellular cytokine staining for CD8^+^ T cells against the HBV peptide pools. *P* values for differences between the vaccine groups are shown above the lines. (**J**) Frequencies of dex^+^CD8^+^ liver lymphocytes (*n* = 4). *P* values are shown within the graph. Data were analyzed by 2-way ANOVA.

To assess whether the vaccines reduced viral loads in animals with long-term exposure to HBV, as is common in patients with CHB, we injected mice with 1 × 10^10^ vg of the AAV8-1.3HBV vector and then vaccinated them 16 weeks later with 1 × 10^10^ vp of AdC6-gDHBV2 or AdC6-HBV2. To further test whether viral load was affected by nonspecific effects of vaccination, an additional group received an AdC6 vector that expresses epitopes of melanoma-associated antigens within gD (AdC6-gDMelapoly). A fourth group was left unvaccinated (Figure 5A). HBV genome copies in sera were tested in 4-week intervals before and after vaccination (Figure 5B). Viral loads after AAV8-1.3HBV injection increased during the following 12 weeks and then stabilized. Mice that received the AdC6-gDHBV2 vaccine showed a significant and sustained decline in titers, with an average reduction of 1.5–2 log_10_. In 1 mouse, HBV genome copies became undetectable. The other groups showed no significant decreases in viral loads. T cell responses tested from the liver at the time of euthanasia by staining with a Pol epitope–specific (FAVPNLQSL-specific) tetramer were markedly higher in AdC6-gDHBV2– than AdC6-HBV2–vaccinated mice (Figure 5C). Both sets of lymphocytes showed high frequencies of cells expressing T-bet (Figure 4D). Splenocytes were tested against the peptide pools and against individual peptides present in the HBV2 sequence. Responses were potent and broad only upon immunization with AdC6-gDHBV2 (Figure 5E). The lack of a more potent response to AdC6-HBV2, as was observed in mice vaccinated 4 weeks after AAV-1.3HBV infection, is noteworthy and may reflect that inclusion of gD in the vaccine insert was able to overcome CD8^+^ T cell burnout upon long-term antigenic exposure.

Even in mice that had been challenged with AAV8-1.3HBV, AdC6-gDHBV2–induced CD8^+^ T cell responses remained remarkably broad, although the specificity profile was strikingly different from that elicited in mice that had not been challenged with the AAV8-1.3HBV vector (Figure 2D). For example, the immunodominant epitope (FAVPNLQSL) that was recognized upon vaccination in unchallenged mice and that forms the basis for both the MHC class I tetramer failed to trigger an IFN-γ response from CD8^+^ T cells of vaccinated mice that had been challenged. To ensure that loss of recognition of this immunodominant epitope was not caused by the longer time interval between testing and vaccination, we repeated the T cell epitope mapping in AAV8-1.3HBV–challenged vaccinated mice 8 weeks after vaccination and again we were unable to detect a response to peptides containing the FAVPNLQSL sequence. Also, while in unchallenged mice responses were mainly directed against the N-terminal part of Pol, in challenged mice T cells preferentially recognized C-terminal sequences of Pol or epitopes within core. This may reflect that CD8^+^ T cells against the more immunodominant epitopes had been rendered unresponsive by the AAV8-1.3HBV challenge.

We repeated the experiment with AdC6-gDHBV2 and AdC6-gDMelapoly (Figure 5F) and saw a significant 2 log_10_ decline of HBV genome copies over the course of 20 weeks following vaccination in the former group (Figure 5G). In this experiment, we tested sera for HBsAg, which initially increased in the AdC6-gDMelapoly group after vaccination and then stabilized, but decreased upon vaccination with AdC6-gDHBV2 by 1–2 log_10_ and in 2 animals HBV genome copies became undetectable (Figure 5H). IFN-γ–producing CD8^+^ T cells against the HBV antigens present in the vaccine were readily detectable in PBMCs and spleens tested 4 or 22 weeks after AdC6-gDHBV2 vaccination, respectively (Figure 5I), and their frequencies tested at 4 weeks after vaccination were inversely correlated with viral loads (Spearman’s *R* = 0.72, *P* = 0.025) and HBsAg levels (Spearman’s *R* = 0.82, *P* = 0.005). Frequencies of CD8^+^ T cells against an immunodominant epitope within Pol were very high in liver, as shown by staining with an MHC class I dextramer that, like the tetramer, was based on peptide FAVPNLQSL (Figure 5J and Supplemental Figure 2A). Additional stains for exhaustion markers and the intracellular transcription factors tested with hepatic lymphocytes from AAV8-1.3HBV–challenged mice injected with the AdC6-gDPolN vaccine indicate that a sizeable percentage of the vaccine-induced dextramer^+^ CD8^+^ T cells did not show increases in exhaustion markers (Supplemental Figure 2B).

## Discussion

Functional cure for CHB has not yet been achieved with current and investigational therapies. Among patients who achieve a cure after pegylated-IFN treatment, viral and HBsAg antigen clearance is accompanied by increases in HBV-specific CD8^+^ T cells (2), indicating that immunological interventions may be able to clear CHB infection.

HBV has mastered evasion of multiple pathways of innate and adaptive immunity, which weakens protective immune responses and promotes progression of CHB (41). In addition, continued presence of large amounts of viral antigens gradually impairs antiviral T cell responses; HBV-specific CD8^+^ T cells over time lose functions, undergo irreversible epigenetic changes, differentiate toward full exhaustion, and undergo apoptosis (11, 42). Immunological interventions thus face several challenges. The epigenetic changes that characterize terminally exhausted T cells are irreversible, thereby blunting the efficacy of checkpoint inhibitors (43). Vaccines that aim to stimulate memory T cells will not expand those that are already exhausted. Vaccines that target naive HBV-specific CD8^+^ T cells are faced with a paucity of such cells in most patients with CHB, as the majority have already been activated previously.

Data presented here suggest that AdC6-gDHBV2 may help overcome some of these challenges. During chronic virus infections, T cells, once activated, differentiate toward exhaustion and in CHB are further impaired by Tregs; both immunosuppressive mechanisms mainly target T cells against immunodominant epitopes. T cells against subdominant epitopes are generally not induced by an acute infection and if they finally become activated during a chronic infection, which is hampered during CHB by the tolerogenic microenvironment of the liver, they are more resistant to exhaustion or suppression (38–40). As we showed previously, vaccines encoding an antigen together with HSV gD induce potent CD8^+^ T cell responses not only to immunodominant, but also subdominant, epitopes (38, 44). Accordingly, the gD-containing HBV vaccine presented here enhances not only the frequency, but also the breadth of CD8^+^ T cell responses, and lowers HBV viral load in a surrogate mouse model of HBV. We observed retention of significant frequencies of vaccine-induced CD8^+^ T cells that remained functionally active, as shown by production of IFN-γ, and maintained expression of T-bet, a transcription factor that regulates cytokine production and that is downregulated in exhausted T cells (11). These results indicate that in this system the HBV-specific CD8^+^ T cells induced by the vaccines in AAV8-1.3HBV–challenged mice did not differentiate toward exhaustion, as was also confirmed by a lack of upregulation of exhaustion markers or the transcription factor TOX1.

It is also of interest to note that the vaccine-induced CD8^+^ T cells’ epitope recognition profile shifted in AAV-1.3HBV–challenged mice. Upon vaccination of unchallenged mice with the AdC6-gDHBV2 vector, most CD8^+^ T cells recognized peptides from the N-terminus of Pol and 8 out of 10 of the highest scoring peptides were part of this sequence. In contrast, in AAV-1.3HBV–challenged mice, most vaccine-induced CD8^+^ T cells responded to peptides from core and the C-terminus of Pol (9 out of the 10 highest scoring peptides), and responses to peptides that had been very potent in unchallenged mice either declined or disappeared. Challenged mice that were vaccinated with AdC6-HBV2 lacked responses to any of the individual peptides. These data again support the concept that gD, as reported previously (38), broadens responses to subdominant epitopes that are less susceptible to impairment by the sustained presence of the AAV vector–delivered HBV antigens. A single i.m. dose of the AdC6-gDHBV2 vector achieves a sustained 2–3 log_10_ cps/mL HBV DNA reduction in mice infected with 1 × 10^9^ or 1 × 10^10^ vg of the AAV8-1.3HBV vector. A significant reduction in viral loads and levels of HBsAg could even be achieved when mice were exposed to HBV for 16 weeks, which, considering the average life span of a mouse, roughly equals 10 human years. Although AdC6-gDHBV2 does not express sequences from HBsAg, it achieved in mice vaccinated 4 or 16 weeks after the initial exposure to HBV a significant reduction in levels of circulating HBsAg, most likely reflecting elimination of the AAV8-1.3HBV–transduced cells by Pol- or core-specific CD8^+^ T cells. CD8^+^ T cell responses induced by an AdC6-HBV2 vaccine, which expresses the same antigen but without gD, are lower and less broad, and accordingly this vaccine is less effective in mice challenged with AAV8-1.3HBV 4 weeks earlier. Delaying vaccination until 16 weeks after challenge further impaired the immunogenicity and efficacy of AdC6-HBV2.

In both of the challenge experiments, frequencies of HBV antigen-specific CD8^+^ T cells inversely correlated with viral loads and levels of circulating HBsAg, indicating that these cells control the infection presumably by eliminating HBV-producing hepatocytes.

Other investigational vaccines have utilized the AAV mouse model to assess HBV load and HBsAg declines. For example, multiple vaccinations with a DNA plasmid vaccine, a protein prime/modified vaccinia virus Ankara (MVA) boost combination regimen, or multiple vaccinations using a human adenovirus–based vaccine, failed to achieve more than a 1 log_10_ cps/mL HBV DNA decline, with minimal HBsAg declines (45–48). Vaccine studies that included HBsAg reported declines in this antigen likely caused by vaccine-induced antibodies, but failed to see marked reduction in viral loads, which requires destruction of virus-producing hepatocytes by CD8^+^ T cells (46, 49).

One challenge that cannot be addressed with the surrogate HBV model based on the AAV8-1.3HBV vector is that it is does not produce cccDNA, which is a key factor for HBV persistence in CHB. In addition, HBsAg, which is produced in excessive amounts during CHB, originates both from cells harboring the viral cccDNA, which continuously produces new viral progeny and HBsAg, and from cells with transcriptionally active integrated HBV genome copies. Such cells could produce HBsAg without expressing Pol and core and would therefore fail to be targeted by CD8^+^ T cells induced by our immunotherapy. Another setback is that the AAV8-1.3HBV vector given i.v. to mice, unlike a natural infection in humans, fails to induce detectable CD8^+^ T cell responses to antigens of HBV so that the vaccine may primarily have activated naive rather than memory T cells, as would be expected upon vaccination of patients with CHB.

Safety of effective therapies for CHB is also a concern. Current immunomodulatory drugs, as well as other checkpoint inhibitors, alone or in combination, are often limited by significant adverse events (50, 51). The genetically encoded gD is only expressed locally at the injection site and regional draining lymph nodes and therefore the potential for serious “off-target” side effects should be low. The potential for improved tolerability and ease of administration via i.m. injection could be advantageous for AdC6-gDHBV2, when used in combination, as a potential functional cure regimen, with other immunomodulators and/or treatments for CHB.

In conclusion, in preclinical studies, a vaccine containing a genetically encoded checkpoint modifier of early T cell activation, administered once i.m., induced increased and broad CD8^+^ T cell responses to HBV core and Pol, which translated into improved preclinical viral load reductions and HBsAg declines, which were strongly inversely correlated with intrahepatic CD8^+^ T cell frequencies. A phase Ib clinical trial of AdC6-gDHBV2 for CHB is currently ongoing and thus far the vaccine has been shown to be well tolerated and to induce HBV-specific T cells even in individuals without detectable responses prior to vaccination (52).

## Methods

### Sex as a biological variable.

Experiments were conducted with male mice, as AAV vectors show higher transduction levels in livers of male than female mice.

### Ethical statement and animal experimentation.

Six-week-old, C57BL/6 mice (Jackson Laboratory) were housed at the Wistar Institute Animal Facility, an AAALAC-accredited Institution. Mice were treated according to approved protocols. Experiments were conducted with groups of 5–10 male mice 2 or 3 times.

### Cell lines.

HEK293 cells (American Type Culture Collection) were maintained in Dulbecco’s modified Eagle’s medium (DMEM) supplemented with 10% fetal bovine serum (FBS, Tissue Culture Biologicals) and antibiotics.

### Antigen selection and vector generation.

Amino acid sequences from core and Pol of HBV genotypes A, B, C, and D were selected from 8,629 publicly available viral genomes and compared for consensus sequences within each genotype using the Shannon Entropy tool hosted by the Los Alamos National Laboratory (https://www.hiv.lanl.gov/content/sequence/ENTROPY/entropy_one.html). Areas of amino acid variations within each genotype were tested using epitope prediction algorithms across multiple HLA haplotypes (http://tools.iedb.org/main/), and the most immunogenic sequences were selected. A segment of core, which has been described to elicit T cells in humans that were associated with viral clearance, was also included (33–36). Initial immunogenicity experiments were performed for core and 2 distinct regions of Pol (C- and N-terminus), each inserted into gD, and placed into an E1-deleted, partial E3–deleted AdC6 vector, which was purified and titrated as described previously (53). Vectors were tested for induction of CD8^+^ T cell responses in mice (43) and based on these responses, we selected the most immunogenic regions of Pol and combined them with core sequences to construct a single vector, thereby avoiding the need for multiple vectors for clinical studies; this construct was cloned into gD and from thereon into the viral molecular clone of AdC6 termed AdC6-gDHBV2. The vector was then produced as described previously (53). The sequences of HBV2 and its insertion site into gD are shown in Supplemental Figure 3, A and B.

Control vaccines expressing HBV2, HIV-gag (without gD), and SARS-CoV-2 nucleocapsid (within gD; termed gDNcap) were created and inserted into AdC6 vectors. Mice immunized with AdC6-gDPolN, expressing the most immunogenic region upon initial testing, and unvaccinated mice were also used as controls in various experiments.

The AAV8-1.3HBV (genotype D) vector, a common tool to study HBV in mice (54), was produced in HEK293 cells by triple transfections with pHelper/pAAV-1.3HBV/pAAV8_capsid_ vectors at a ratio of 1:0.5:0.5. AAV vectors were purified from cell lysate by gradient centrifugation and dialyzed against PBS. AAV titers were determined by SYBR Green (Thermo Fisher Scientific) qPCR assays with insert-specific primers (Forward: TGAGAGGCCTGTATTTCCCTGC; Reverse: AACCCCGCCTGTAACACGAG). Vg copies/mL were calculated based on cycle threshold (Ct) values and a plasmid-derived standard curve.

### Protein production by adenoviral vectors.

The AdC6 vectors were tested for protein expression upon transfection of HEK293 cells with 1000 vp/cell for 48 hours at 37°C. Cell lysate was resolved in a 12% SDS-PAGE gel and transferred to a polyvinylidene difluoride (PVDF) membrane (Merck Millipore), which was blocked in 5% powdered milk overnight at 4°C. The gD antibody diluted 1:1000 (clone PA1-30233, Invitrogen) was added for 1 hour at room temperature. Membranes were washed with 1× TBS-T and incubated with HRP-conjugated goat anti-rabbit secondary IgG for 1 hour at room temperature. Membranes were also probed with a mouse monoclonal IgG antibody against β-actin (sc-47778, Santa Cruz Biotechnology). Protein was visualized on membranes with Super Signal West Pico Chemiluminescent (Thermo Fisher Scientific) (Supplemental Figure 3C). Lysates of cells infected with the AdC6-HBV2 vectors, for which antibodies are not available, were analyzed by LC-MS/MS (Supplemental Figure 3D).

### Vaccination and infection of mice.

AdC6 vectors were diluted in sterile saline to 200 μL and injected i.m. into the hind legs of mice. The AAV8-1.3HBV vector was diluted in sterile saline and injected at a volume of 300 μL into the tail vein.

### Preparation of PBMCs.

Blood from the saphenous vein was collected into Liebowitz’s L-15 medium (Thermo Fisher Scientific) with 4% sodium carbonate. PBMCs were purified by Ficoll-Paque Plus (GE Healthcare) gradient centrifugation for 30 minutes at 400*g*. Cells were washed and seeded into round-bottom 96-well plates (0.2 × 10^6^ to 1 × 10^6^ cells per well).

### Lymphocyte collection from livers and spleens.

Single-cell suspensions were generated by mincing spleens in Liebowitz’s L-15 medium followed by passing cells through a 70-μm filter (Thermo Fisher Scientific). Red blood cells were lysed (1× RBC lysis buffer, eBioscience). Cells were washed with DMEM with 10% FBS. To isolate hepatic lymphocytes, livers were cut into fragments and treated with 2 mg/mL collagenase P, 1 mg/mL DNase I (both from Roche), and 2% FBS in Liebowitz’s L-15 under agitation for 1 hour. Liver fragments were homogenized, filtered through 70-μm strainers, and lymphocytes were purified by Percoll gradient centrifugation and washed with DMEM with 10% FBS.

### In vitro stimulation of lymphocytes.

Lymphocytes were stimulated with pools of peptides or individual peptides. Peptides diluted according to the manufacturer’s instructions were 15 amino acids in length and overlapped by 10 amino acids with the adjacent peptides. For stimulation, approximately 1 × 10^6^ lymphocytes plated in medium containing 2% FBS and GolgiPlug (BD Bioscience) at 1.5 μL/mL were cultured with peptides each present at a final concentration of 2 μg/mL for 5 hours at 37°C in a 5% CO_2_ incubator. Control cells were cultured without peptides.

### Intracellular cytokine staining and analyses by flow cytometry.

Cells were incubated with anti-CD8–APC (clone 53-6.7, BioLegend), anti-CD4–BV605 (clone RM4-5, BioLegend), anti-CD44–Alexa Flour 700 (clone IM7, BioLegend), and Live/Dead Violet dye (Thermo Fisher Scientific) at 4°C for 30 minutes in the dark. Cells were washed with PBS and fixed and permeabilized with Cytofix/Cytoperm (BD Biosciences) for 20 minutes. Cells were incubated with an anti–IFN-γ–FITC antibody (clone XMG1.2, BioLegend) at 4°C for 30 minutes in the dark. In some experiments, an anti-TOX1–PE antibody (clone TXRX10, eBioscience) was added. Cells were washed and fixed in a 1:3 dilution of BD Cytofix fixation buffer (BD Pharmingen) and analyzed using a FACSCelesta (BD Biosciences) and DiVa software. Postacquisition analyses were performed with FlowJo (TreeStar). Data represent percentage IFN-γ–producing CD8^+^ or CD44^+^CD8^+^ cells upon peptide stimulation. Background values obtained for the same cells cultured without peptide(s) were subtracted.

### Tetramer/dextramer staining.

Lymphocytes were stained with Live/Dead Violet, anti-CD8–PerCPCy5.5, anti-CD44–Alexa Flour 700, anti–PD-1–BV605 (clone 29F.1A12, BioLegend), anti–LAG-3–BV650 (clone C9B7W, BioLegend), and an APC-labeled MHC class I tetramer (NIH Tetramer Facility) or an APC-labeled dextramer (Immudex), both corresponding to amino acids 396–404 (FAVPNLQSL) of Pol, at 4°C for 30 minutes in the dark. In some experiments, additional surface stains for anti–PD-1–BUV395 (clone Ly101, BD Biosciences), anti-CTLA–4-PE (clone Uc10-4B9, BioLegend), anti–LAG-3–BV650 (clone C9B7w, BioLegend), or intracellular stains for EOMES–Alexa Fluor 488 (clone c77258, BD Bioscience) or T-bet–BV785 (clone 4B20, BioLegend) were added. Cells were washed and analyzed using a FACSCelesta and DiVa software.

### Titration of HBV genomes.

Blood was harvested, sera were prepared, and DNA was extracted using a DNeasy Blood & Tissue Kit (Qiagen). The qPCR, which for each run contained serially diluted plasmids expressing the HBV sequence to provide a standard curve, was performed in a total volume of 20 μL, including 1 μL of serum per PCR well. The following primers were used for amplification: forward, 5′-TGAGAGGCCTGTATTTCCCTGC-3′ and reverse, 5′-AACCCCGCCTGTAACACGAG-3′. Ct values of the standard curves were used to determine copy numbers per μL. Data were adjusted to 1 mL of serum. Water and sera from naive mice served as controls.

### ELISA for HBsAg.

Levels of HBsAg were determined using a 1:2000 serum dilution with the HBsAg ELISA Kit (KA0286, Abnova Corp). Positive and negative controls provided by the kit as well as sera from naive mice were included.

### ELISA for antibodies against HBsAg.

IgG antibodies against HBsAg were tested in sera from mice injected with 1 × 10^10^ vg of AAV8-1.3HBV. Sera were harvested 12 weeks after immunization and tested at 1:100 and 1:400 dilutions on plates coated with 5 μg/mL of recombinant HBsAg (Creative Diagnostics). An anti-HBsAg antibody was used as a positive control. A 1:1500 dilution of the secondary antibody (goat anti–mouse IgG, Sigma-Aldrich) and KPL DEA phosphatase substrate (SeraCare) served to visualize antibody binding. OD values obtained with wells that received buffer rather than sera were subtracted from the OD values obtained with experimental samples.

### Statistics.

Two-group comparisons used Mann-Whitney tests. Multiple comparisons used 2-way ANOVA. HBV DNA viral loads and HBsAg titers in sera were assessed before and after vaccination and relationships between viral parameters and frequencies of IFN-γ–producing CD8^+^ T cells were assessed by Spearman’s rank correlations. In the box-and-whisker plots, the line in the box is the median, the box bounds represent the IQR, and whiskers the minimum and maximum.

### Study approval.

This research adhered to the policies and guidelines of ARRIVE and was approved by the Institutional Animal Care and Use Committee of the Wistar Institute.

### Data and materials availability.

All data, code, and materials used in the analysis are available upon request to any researcher for purposes of reproducing or extending the analysis. The raw data used for the figures are available in the supplemental Supporting Data Values file. Transfer of some of the material may require a transfer agreement between the institutions. All data but for one confirmatory study listed as data not shown are available in the main text or the supplemental materials.

## Author contributions

HCJE conceptualized the study, provided project administration, and supervised the study. MH, MN, RA, AC, DN, ZX, and XZ developed methodology. MH carried out the investigation. HCJE, ADL, and SLC wrote the original draft of the manuscript, which was reviewed and edited by MN, HCJE, ADL, and SLC.

## Supplementary Material

Supplemental data

Supporting data values

## Version 1 09/03/2024

In-Press Preview

## Version 2 11/08/2024

Electronic publication
