# Supplementary figures and images for: A therapeutic HBV vaccine containing a checkpoint modifier enhances CD8+ T cell and antiviral responses
Source: JCI Insight. 2024 Nov 8;9(21):e181067. doi: 10.1172/jci.insight.181067 (PMC11601613; doi:10.1172/jci.insight.181067)

A

A

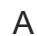

A

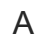

A

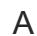

Figure S2

**A Tetramer stain**

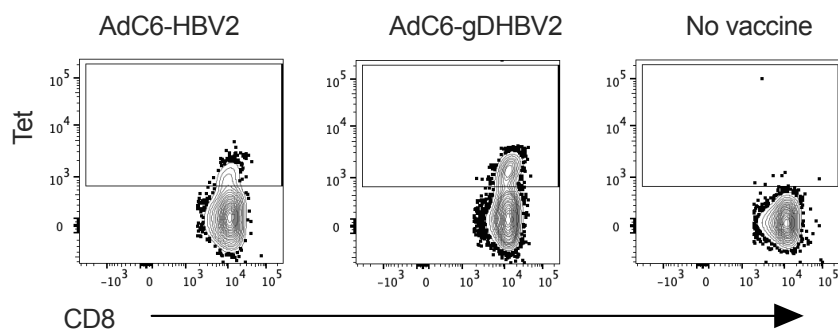

**B Dextramer stain**

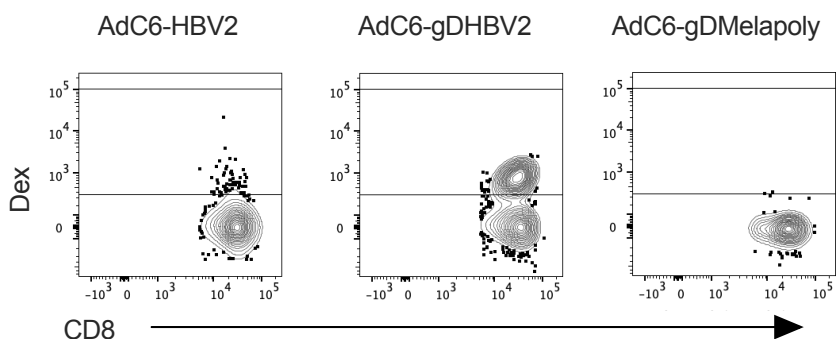

**C**

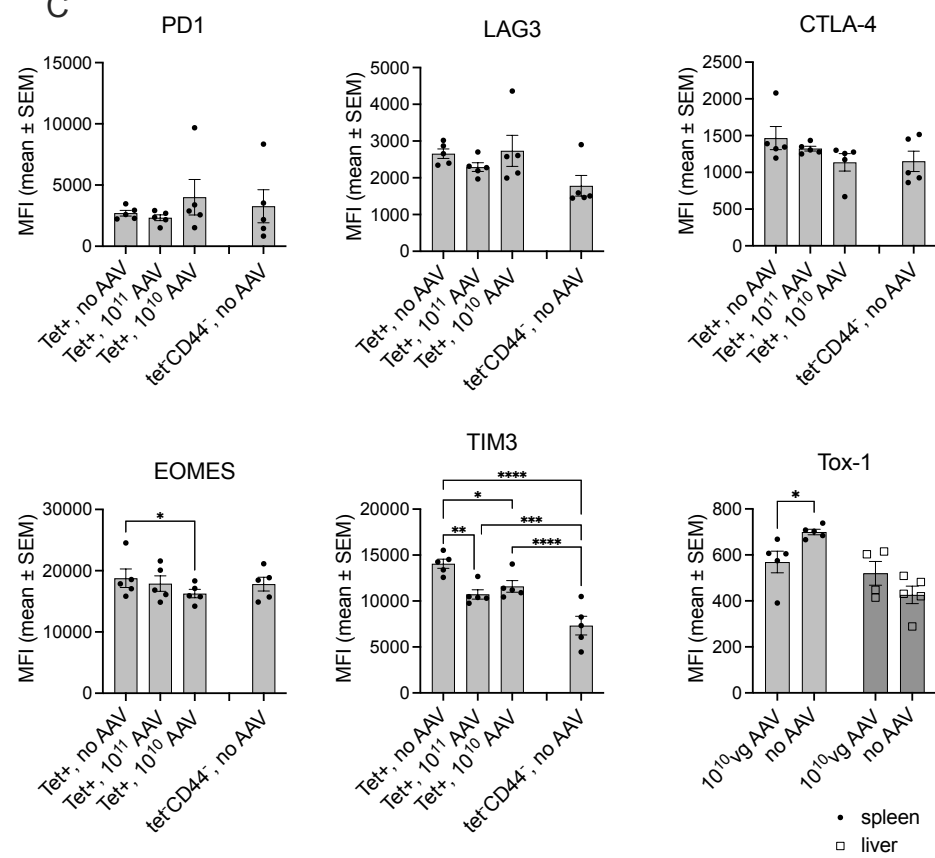

Figure S3

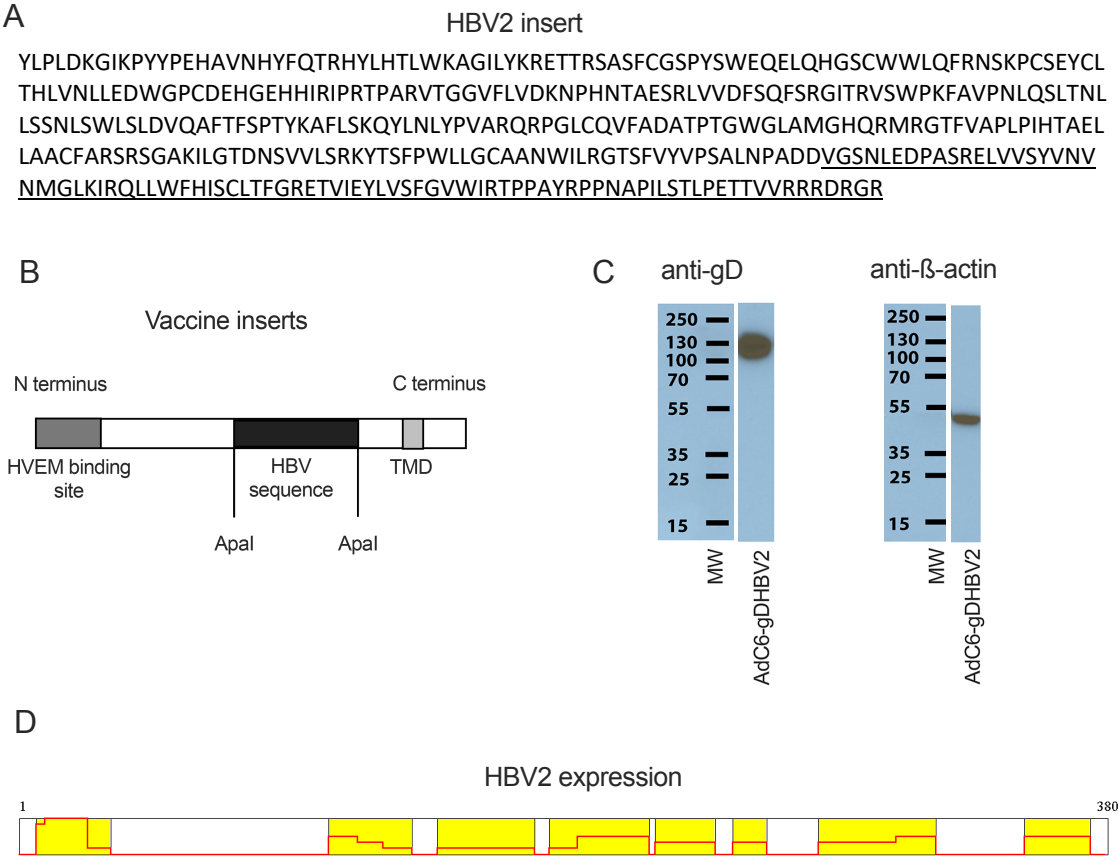

Supplement: Supplemental data [file jciinsight-9-181067-s065.pdf]
